
JOURNAL INFORMATION
==============================
NLM Title Abbreviation: ScientificWorldJournal
Journal ID: ScientificWorldJournal
NLM Journal ID: 101131163
Journal ID: TSWJ

The Scientific World Journal

ISSN: 2356-6140
EISSN: 1537-744X
Publisher: Wiley

ARTICLE INFORMATION
==============================
PMCID: PMC6083891
DOI: 10.1100/tsw.2001.406
Article ID: 126989
Article version: 1
Subjects: Extended Abstract

Effect of CGRP and Related Peptides in the Microcirculation

Brain S. D.
Chu D. Q.
Centre for Cardiovascular Biology and Medicine, New Hunt’s House, Guy’s Campus, King’s College, London, SE1 1UL, UK
Publication date: 2001
Electronic publication date: 2001 Sep 24
Volume: 1
First page: 34
Last page: 34
Copyright: Copyright © 2001 S. D. Brain and D. Q. Chu.
Copyright year: 2001
License: This is an open access article distributed under the Creative Commons Attribution License, which permits unrestricted use, distribution, and reproduction in any medium, provided the original work is properly cited.
License URL: https://creativecommons.org/licenses/by/3.0/

==============================
4th International Meeting on Calcitonin Gene-Related Peptide (CGRP)
*TheScientificWorld* (2001) 1(S1), 34
ISSN 1532-2246; DOI 10.1100/tsw.2001.406

### EFFECT OF CGRP AND RELATED PEPTIDES IN THE MICROCIRCULATION

S.D. Brain and D.Q. Chu
Centre for Cardiovascular Biology and Medicine, New Hunt's House, Guy's Campus, King's College, London, SE1 1UL, U.K.

We are interested in the potential of CGRP and adrenomedullin (ADM) as proinflammatory peptides and have compared the ability of CGRP and ADM to modulate microvascular and nociceptive (thermal hyperalgesic) responses in rat skin. Vasodilator activity was assessed by laser Doppler flowmetry, inflammatory oedema by the extravascular accumulation of i.v.-injected radiolabelled albumin, and neutrophil accumulation by tissue myeloperoxidase. Hyperalgesia was assessed by a thermal hyperalgesimeter in paw skin.

ADM (30 to 300 pmol) was threefold less potent than CGRP as a vasodilator. CGRP (30 pmol) potentiated oedema formation induced by mediators of increased microvascular permeability, as expected ($p < 0.01$). However, ADM (30 to 100 pmol) was without a potentiating effect, although ADM (300 pmol) was effective ($p < 0.01$). By comparison ADM (100 pmol) potentiated cell accumulation induced by the neutrophil attractant IL1-β, whereas CGRP (30 pmol) did not. Thus ADM, like CGRP, has the ability to potentiate inflammatory events in the microcirculation albeit with apparent differential potencies, that are intriguing and possibly related to actions via different receptors. However, despite proinflammatory activity, neither CGRP nor ADM appears to mediate thermal hyperalgesic activity in skin.

The comparative activities of endogenous CGRP and ADM is more difficult to determine but our results so far suggest that ADM mRNA is expressed at microvascular sites in skin as early as 4 h in inflammatory responses, where the increased blood flow can be inhibited by $CGRP_{8-37}$. The relative contribution of CGRP and ADM is under study.

This study was funded by the British Heart Foundation.
